# Tear cytokine and chemokine analysis and clinical correlations in evaporative-type dry eye disease

**Published:** 2010-05-19

**Authors:** Amalia Enríquez-de-Salamanca, Evangelina Castellanos, Michael E. Stern, Itziar Fernández, Ester Carreño, Carmen García-Vázquez, Jose M. Herreras, Margarita Calonge

**Affiliations:** 1 Ocular Surface Group, IOBA-University of Valladolid, Valladolid, Spain; 2 Statistics Unit, IOBA-University of Valladolid, Valladolid, Spain; 3 CIBER-BBN, Valladolid, Spain; 4 Allergan Inc., Irvine, CA

## Abstract

**Purpose:**

Inflammatory molecules have been demonstrated in the tear film of patients with severe dry eye disease (DED). However, little attention has been paid to the most frequent moderate forms of DED. This study analyzes tear cytokine levels and their clinical correlations in patients with moderate evaporative-type DED due to meibomian gland disease (MGD).

**Methods:**

Twenty three evaporative-type DED patients (46 eyes) of mild-to-moderate intensity and nine healthy subjects (18 eyes) were recruited. Two symptom questionnaires were self-answered and multiple DED-related clinical tests were performed. Unstimulated tears from each eye were isolated and were not pooled. Levels of 15 cytokines and chemokines were measured by multiplex bead analysis, compared with control levels, and correlated with clinical tests.

**Results:**

Fourteen out of the 15 molecules were reliably detected in 1 μl of unstimulated tears from DED patients. Epidermal growth factor (EGF), fractalkine/CX3CL1, interleukin (IL) 1-receptor antagonist (Ra), IL-8/CXCL8, interferon inducible protein (IP)-10/CXCL10, and vascular endothelial growth factor (VEGF) were found in 94%–100% of samples; IL-6 in 65% (significantly more detected in older patients); IL-1β, interferon gamma (IFN-γ), and IL-10 in 30%–48%; IL-17 in 13%; granulocyte macrophage colony stimulating factor (GM-CSF), IL-13, and tumor necrosis factor alpha (TNF-α) in 2%–9%; and IL-5 was never detected. EGF, fractalkine/CX3CL1, IL-1Ra, IP-10/CXCL10, and VEGF levels were significantly increased compared to normal controls. Pain was correlated with IL-6 and IL-8/CXCL8. Tear break-up time correlated inversely with IL1-Ra. Schirmer test and tear lysozyme levels negatively correlated with IL-1Ra, IL-8/CXCL8, fracktalkine/CX3CL1, IL-6, IP-10/CXCL10, and VEGF had the same tendency. Conjunctival staining correlated negatively with EGF and positively with IL-6.

**Conclusions:**

In this sample of moderate evaporative-type DED patients, five inflammatory molecules were elevated. Fracktalkine was demonstrated to be present and elevated in tears in human DED. IL-1Ra, IL-6, IL-8/CXCL8, and EGF levels correlated with pain and with clinical parameters measuring tear stability, tear production or ocular surface integrity. These results suggest that inflammation plays a role not only in severe DED but also in moderate evaporative DED.

## Introduction

Dry eye disease (DED) is a major and increasing health-care problem due to its high prevalence and capacity to affect patients’ quality of life, work-related issues, and health-care resources [1-3]. DED was recently redefined [4] as a multifactorial disease of the tears and ocular surface that results in symptoms of discomfort [5-7], visual disturbance [8-10], and tear film instability [11-13] with potential damage to the ocular surface. It is accompanied by increased osmolarity of the tear film [14-17] and inflammation of the ocular surface [18,19].

To diagnose DED, a combination of symptom questionnaires and clinical tests is recommended [20]. These clinical tests evaluate tear clearance (production and elimination), tear stability, and ocular surface integrity. There are some other more specialized tests that may eventually be introduced into clinical practice such as tear osmolarity [17], conjunctival cytology [21], visual function [22], and confocal microscopy [23]. It is universally recognized that symptoms and clinical signs do not correlate well in DED, especially in mild-to-moderate forms [6,24-29]. Therefore, additional clinical tests that demonstrate the presence of and characterize the type of inflammation would be of invaluable help, especially if those tests correlate with clinical signs and symptoms. This would be a major advance in DED clinical trials, where evaluation endpoints often fail to adequately show therapy benefits, partially explaining the scarcity of therapies for DED.

Increased levels of several inflammatory cytokines have been correlated with clinical parameters in aqueous-deficient DED and in evaporative-type, rosacea-related DED [18,30-34]. Therefore, tear cytokine levels are already considered as potential markers of inflammation in DED. However, the majority of patients with DED have moderate forms of the disease [35] for which studies are more limited and some have failed to show increased levels of cytokines that were elevated in more severe forms [36,37]. The aim of the present study was to measure a wide panel of cytokines and chemokines in tears from evaporative-type DED due to meibomian gland disease (MGD) of moderate intensity. We then compared the levels of these inflammatory mediators with controls and correlated the levels with clinical signs and symptoms.

## Methods

### Patients and healthy controls

A total of 23 consecutive new patients (7 males, 16 females; mean age ±standard deviation [SD] 57.7±12.5 years, range 28–79 years) referred to the University of Valladolid-IOBA (Institute of OphthalmoBiology), Valladolid, Spain, for long-standing dry eye-related symptoms were recruited. Inclusion criteria were as follows: dry eye-related symptoms for at least the previous 12 months, no topical therapies other than artificial tears for the previous 3 months, moderate intensity of symptoms as defined by a score less than half of the total possible value on the symptoms of discomfort questionnaire (SODQ) and the Symptom Assessment in Dry Eye (SANDE) questionnaire. Additionally, each prospective patient had a final diagnosis of evaporative-type of DED due to MGD as defined by typical lid margin changes and meibomian secretion alteration, abnormal tear breakup time (TBUT), normal tests evaluating tear production, and mild-to-negative vital ocular surface staining. Exclusion criteria included any previous or present ocular disease other than DED, surgery or contact lens wear, any systemic disease other than hypertension or hypercholesterolemia which was under control with no need of drugs, such as systemic therapies including antihistamines, anticholinergics, antihipertensives known to affect tear dynamics, or ocular therapies other than artificial tears. Nine healthy volunteers (6 females and 3 males, mean age ±SD 33.1±8 years, range 25–51 years) constituted the control group. These subjects were healthy, were not pregnant, were not under any medication, had no previous or present history of ophthalmic disease, did not have any ocular symptoms, and were not contact lens users. These healthy volunteers had Schirmer’s test 1, lysozyme tear levels, corneal and conjunctival staining, and SODQ score within normal limits. Written consent was obtained from all subjects after explanation of the nature and possible consequences of the study. The study was approved by the University of Valladolid Ethics Committee and followed the Tenets of the Declaration of Helsinki.

### Clinical Examination

Clinical evaluations were performed following the sequence shown in Table 1. At least 10 min were allowed between tests. Clinical evaluations were always done by authors E.C. and M.C. In case of discrepancies on subjective tests, an agreement was reached by averaging the score judged by each clinician. All tear samples were taken by the same clinician (E.C.).

**Table 1:** Questionnaires and clinical tests for dry eye disease (DED) patients.

**Questionnaires and diagnostic tests (normal range or cut off point)**	**Eye**	**Mean±SEM**
SODQ (0-32)		11.57±5.58
SANDE Severity (0-100); Intensity (0-100)		43.04±30.10; 48.78±37.41
Tear meniscus height (>0.3 mm)	OD	1.90±0.80
	OS	1.90±0.80
Phenol red thread test (10 mm; 15 sec)	OD	19.86±5.88
	OS	22.38±5.95
TBUT (>10 sec)	OD	4.48±3.13
	OS	4.22±2.66
Corneal fluorescein staining (0-5)	OD	0.83±1.79
	OS	0.81±1.77
Conjunctival rose bengal staining (0-5)	OD	1.26±1.43
	OS	1.52±1.44
Tear lysozme (>1000 μg/ml)	OD	2872.52±2783.10
	OS	3227.52±2239.95
Schirmer-1 test (>5 mm; 5 min)	OD	16.70±8.44
	OS	17.35±8.86
Lid margin changes /Quality of meibomian gland secretion (0-4)	OD	3.33±0.73
	OS	3.48±0.75

Questionnaires and tests were given in the order listed above. DED; Dry eye disease; SEM: standard error of the means; SODQ: symptoms of discomfort questionnaire; SANDE: symptom assessment in Dry Eye; TBUT: tear breakup time; OD: right eye; OS: left eye.

#### Dry eye-related symptomatology

Dry eye-related symptoms were evaluated with two questionnaires. The SODQ included 8 questions, each one scoring from 0 to 4, to yield a total score of 32 regarding dryness, sandy/gritty feeling, burning/stinging, pain, itching, sensitivity to light, and blurred vision [38]. The SANDE questionnaire evaluated both dry eye intensity and frequency by using a 100 mm visual analog scale [39].

#### Tear sample collection

Tear collection was performed before any other test and with a minimum of 10 min after the patient answered the two symptom questionnaires. Unstimulated tear samples were collected non-traumatically from the external canthus of open eyes, avoiding additional tear reflex as much as possible. Glass capillary micropipettes (Drummond, Broomall, PA) were used to collect 1 μl of tears. Each sample was then diluted 1:10 in a sterile collection tube containing ice-cold Beadlyte® Cytokine Assay Buffer (Upstate-Millipore, Watford, UK). Tubes with tear samples were kept cold (4 °C) during collection, and stored at −80 °C until assayed. The samples were obtained from right eye (OD) and left eye (OS) of each individual, and were not pooled.

#### Tear production and stability

Tear production was assessed with four different tests: tear meniscus height (TMH), Schirmer’s test, phenol red thread test, and tear lysozyme level assay. TMH was measured after fluorescein staining [40] using the slit lamp with a blue cobalt light. The Schirmer-1 test [41] was performed by placing one sterile strip (Schirmer Tear Test Strips, 5×35 mm; Alcon Laboratories, Inc., Fort Worth, TX) in the lateral canthus of the inferior lid margin of both eyes without topical anesthesia. Subjects were asked to maintain their eyes closed during the test, and the length of wetting was measured in millimeters after 5 min. The phenol red thread test (Zone Quick Test; Menicon Ca, Ltd., Nagoya, Japan) was achieved by placing the thread over the lateral canthus of the eye and read after 15 s. Tear lysozyme concentration [42] was determined by the *Micrococcus lysodeikticus* (ATCC 4698, M3770; Sigma-Aldrich, St. Louis, MO) agar diffusion assay in Mueller Hinton agar plates (Bio Merieux, Marcy l’Etoile, France). A 5-mm diameter Resma filter paper disc were gently applied to the inferior conjunctival fornix of each eye for 1 min with eyes closed. Samples were kept at 20 °C until processed. Each filter paper was left in the plate with the *Micrococcus lysodeikticus* inoculum (2×10^6^ CFU/ml) suspension gel, and the inhibition hallus was measured after 24 h. Lysozyme concentration was calculated from a standard curve of the inhibition hallus generated with several concentrations of commercial lysozyme (ATCC 4698, L6876; Sigma-Aldrich).

Tear stability was evaluated by tear break-up time (TBUT) test. Fluorescein strips previously wetted with 0.9% sodium chloride were gently applied to the inferior fornix. The time lapse between instillation of fluorescein and the appearance of the first randomly distributed dry spot was measured after three blinks, and the mean of three measurements was recorded [43].

#### Ocular surface integrity

Corneal and conjunctival integrity were evaluated with fluorescein (Fluorets; Chauvin, Aubenas, France) and rose Bengal (Akorn, Inc., Buffalo Grove, IL) strips, respectively. Following the Oxford Scheme (score 0–5) [44] for grading, corneal and conjunctival staining were scored in each of the five different areas of the eye: central, inferior, superior, nasal, and temporal. Total fluorescein and total rose Bengal scores were calculated as the sum of scores of each of the five areas.

#### Quality of meibomian gland secretion

Meibomian gland function was evaluated last using the slit lamp. Lid changes were recorded to aide in the diagnosis. However, these were not further analyzed or used for correlations with other data. The quality of meibomian secretion, determined after expressing the glands, was judged as clear (0), cloudy (1), granular (2), and solid (3) [45].

### Analysis of cytokine/chemokine concentration

Cytokine and chemokine levels in tear samples were determined by a 15× multiplex bead analysis (Linco-Millipore, Watford, UK) using a Luminex IS-100 (Luminex Corp., Austin TX). This kit detects free, total, and non-degraded forms of the cytokines/chemokines included. Levels of interleukin (IL)-1β, IL-5, IL-6, IL-8/CXCL8, IL-10, IL-13, IL-17, interferon inducible protein (IP)-10/CXCL10, tumor necrosis factor (TNF)-α, vascular endothelial growth factor (VEGF), interferon (IFN)-γ, IL-1 receptor antagonist (IL-1Ra), fractalkine/CX3CL1, granulocyte-monocyte colony stimulating factor (GM-CSF), and epidermal growth factor (EGF) were measured. Ten microliters of sample were incubated with antibody-coated capture beads for 2 h at 20 °C. Washed beads were further incubated with biotin-labeled anti-human cytokine antibodies, followed by streptavidin-phycoerythrin incubation. Standard curves of known concentrations of recombinant human cytokines were used to convert fluorescence units to cytokine concentration (pg/ml). Minimum detectable concentrations (in pg/ml) were IL-1β=0.19, IL-1Ra=10.97, IL-5=0.12, IL-6=0.79, IL-8/CXCL8=0.32, IL-10=0.41, IL-13=4.06, IL-17=0.25, EGF=4.47, Fractalkine/CX3CL1=4.27, GM-CSF=0.23, IFN-γ=0.55, IP-10/CXCL10=1.14, TNF-α=0.22, and VEGF=4.10. Data were stored and analyzed using the Bead View Software (Upstate-Millipore). Cytokine levels in control normal tears were determined similarly, by using a 22-plex (Upstate-Millipore), and a 7-plex (Linco-Millipore) cytokine/chemokine Luminex commercial assays [46]. EGF, fractalkine/CX3CL1, IL-1Ra, IP-10/CXCL10, and VEGF levels were the only molecules that were determined using the same reagents for both DED and control tears. Therefore, to assure identical assay results, only these cytokines and chemokines were compared statistically between DED and control subjects.

### Statistical analysis

Data are presented as means±standard error of the means (SEM), unless otherwise specified in the text. Statistics were analyzed using the SPSS software package (SPSS 15.0 for Windows; SPSS, Chicago, IL) by a licensed statistician (I.F.). The nonparametric Mann Withney U test was used for comparisons of two independent sample groups. To quantify correlations between cytokine/chemokine levels and clinical parameters, the Spearman ranked correlation coefficient (rho) was determined along with 95% confidence bands. Two-sided P-values of ≤0.05 were considered statistically significant.

## Results

### Diagnostic clinical tests

As determined by the selection criteria, the patients in this study had moderate dry eye symptoms based upon the SODQ and SANDE questionnaire, scoring less than 50% of the highest possible values (Table 1). A large panel of tests was given to exclude the possibility that an aqueous-deficient component was contributing to the disease, thus ensuring that DED for our patients could be attributed only to an evaporative-component. Measurements of tear meniscus height, Schirmer test, phenol red thread test, and tear lysozyme concentration were all within normal limits, confirming that these DED patients suffered predominantly from evaporative loss of tears. Additionally, both corneal and conjunctival staining scores were low, denoting little ocular surface damage.

In contrast, TBUT results were below the accepted normal limits (Table 1) denoting tear instability due to excessive evaporation [47], the main consequence of MGD on the tear film. Lid margin changes were consistent with MGD and the quality of secretion was never normal. Based upon results of the clinical examinations, all of the patients were classified as having evaporative-type DED due to MGD of mild-to-moderate intensity.

### Tear concentrations of cytokine and chemokines in DED patients

**Figure 1:**
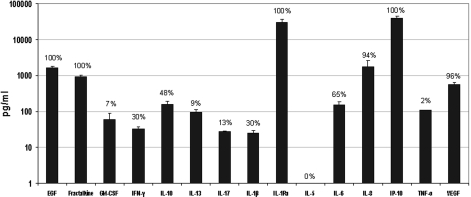
Cytokines and chemokines in tears of DED patients. Fourteen out of the 15 molecules analyzed were detected. EGF, Fractalkine/CX3CL1, IL-1Ra, IP-10/CXCL10, VEGF, and IL-8 were the more detected molecules in evaporative-type DED tear samples and were also the ones with the highest concentration. Data are presented on a logarithmic scale and bars represent values for OS and OD together. Percents of patients having detectable amounts are shown at the top of each bar. EGF, epidermal growth factor; IFN-γ, interferon gamma; GM-CSF, granulocyte-macrophage colony-stimulating factor; IL, interleukin; IL-1Ra, interleukin 1 receptor antagonist; TNFα, tumor necrosis factor alpha; IP-10, interferon inducible protein 10; VEGF, vascular endothelial growth factor.

Cytokine and chemokine levels were individually measured in OD and OS tear samples of all patients. Of the 15 molecules analyzed, EGF, fractalkine/CX3CL1, IL-1Ra, and IP-10/CXCL10 were detected in all samples (Figure 1). For IL-1Ra and IP-10/CXCL10, 52% and 74%, respectively, of samples had values above the highest point of the standard curve. In these two cases, we assigned the value as the highest detectable concentration for that assay. IL-8/CXCL8 and VEGF were detected a 95.7% and a 93.5%, respectively. IL-6 was detected in 65% of samples, and IL-10 was detected in 48% of patients. The following cytokines and chemokines were detected in 30% or fewer of the patients: IFN-γ (30%), IL-1β (30%), IL-17 (13%), IL-13 (9%), GM-CSF (7%), and TNFα (2%). IL-5 was the only molecule not detected in any sample (Figure 1). All six of the most frequently detected cytokines and chemokines were also the ones with the highest concentrations.

**Figure 2:**
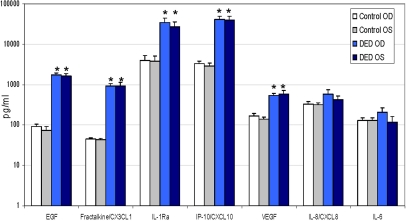
Comparison of tear cytokine/chemokine levels in DED patients with control subjects. EGF, Fractalkine/CXC3L1, IL-1Ra, IP-10/CXCL10, and VEGF levels were significantly increased in evaporative-type DED tear samples compared to controls. Values are presented on a logarithmic scale. EGF, epidermal growth factor; IL, interleukin; IL-1Ra, interleukin 1 receptor antagonist; IP-10, interferon inducible protein 10; VEGF, vascular endothelial growth factor. OD, right eye; OS, left eye. *p<0.05, DED tears compared to corresponding controls.

EGF, fractalkine/CX3CL1, IL-1Ra, IP-10/CXCL10, and VEGF levels in tears of DED patients were compared to control tear levels determined in 9 healthy subjects (18 eyes). Each of the molecules was significantly increased in the DED patients compared to controls (Figure 2). IL8/CXCL8 and IL-6 values in DED tears were similar to those found in control tears, although we did not compare them statistically because they were analyzed using different commercial kits. When cytokine/chemokine levels between OD and OS were compared, only for IL-6 was there a significant difference, although the values were quite similar (OD: 209.9±56.6 pg/ml; OS: 115.28±47 pg/ml; p=0.033).

### Correlation analyses between tear cytokine/chemokine levels and clinical signs and symptoms

We performed correlation analyses between cytokine/chemokine levels and all signs and symptoms for the seven molecules detected in more than 50% of DED patients (Figure 2): EGF, fractalkine/CX3CL1, IL-1Ra, IL-8/CXCL8, IP-10/CXCL10, VEGF, and IL-6. Because we did not pool tear samples, each correlation between the cytokine and chemokine levels and the signs and symptoms maintained OD and OS laterality.

#### Correlation with age and gender

**Figure 3:**
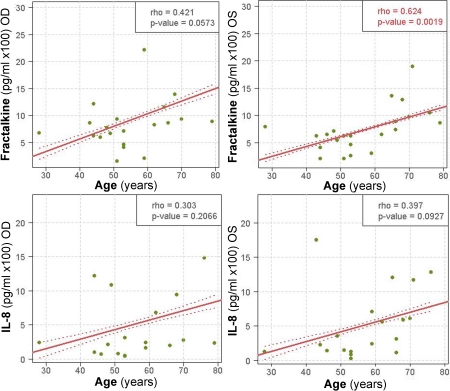
Fractalkine/CX3CL1 and IL-8/CXCL8 levels correlation with age in DED patients. Fractalkine/CX3CL1 and IL-8/CXCL8 levels correlated positively with age in evaporative-type DED tear samples. Spearman ranked correlation test was used and the strength of correlation between variables was determined by the Spearman's rho correlation coefficient. Dotted lines indicate the 95% confidence bands. IL, interleukin; OD, right eye; OS, left eye.

There was a significant positive association with age for fractalkine/CX3CL1 levels in OS (p=0.0019) and borderline significant in OD (p=0.0573; Figure 3); IL-8/CXCL8 levels in both eyes had the same tendency, almost reaching significance for OS (p=0.09). Those patients for whom IL-6 was detected were significantly older, 66±6 years than the 35% of patients for whom IL-6 could not be detected, 53±3 years (p=0.05). There were no significant correlations between cytokine/chemokine levels and gender except for OD IL-8/CXCL8 levels being significantly higher in males than females (p<0.05).

#### Correlation with symptoms

**Figure 4:**
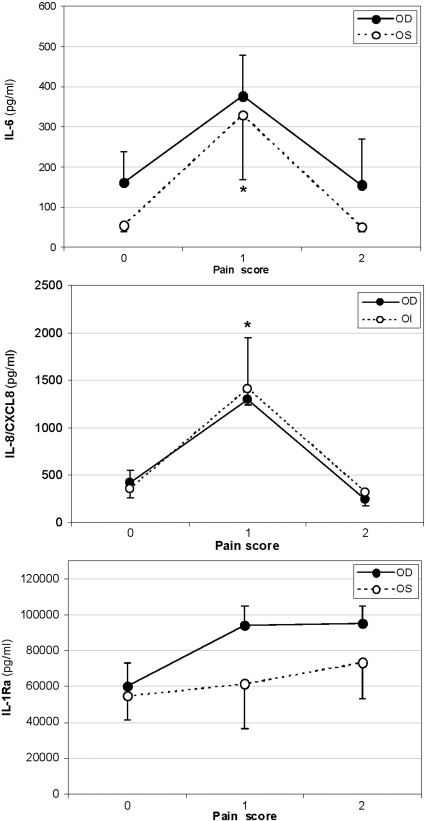
Correlation studies with SODQ pain score. IL-6 and IL-8/CXCL8 were significantly increased for pain score 1 compared to pain scores 0 and 2 (OS *p=0.003 for IL-6; *p=0.027 for IL-8/CXCL8; OD p-values were not significant but followed the same trend as OS). IL-1Ra levels increased with pain in both eyes but did not reach statistical significance. IL, interleukin; OD, right eye; OS, left eye.

No correlations were found between any molecule detected in tears with the global scores for either the SODQ or SANDE questionnaire. However, some significant correlations were obtained when each of the items evaluated in SODQ questionnaire were individually compared with cytokine levels. Thus, when pain was analyzed, IL-6 and IL-8/CXCL8 levels in OS were significantly higher in patients scoring 1 versus those scoring 0 or 2 (Figure 4). OD values had the same tendency, although the correlations were not significant. IL-1Ra levels increased with pain score in both eyes but did not reach statistical significance (Figure 4). Regarding sandy/gritty feeling, for IL-6 there was a tendency toward positive correlation but the results were not statistically significant for either eye. IL-6 levels in OS also showed a tendency to correlate with blurred vision (p=0.077), with higher levels for patients that scored 3–4 of the scale in which 4 is the maximum.

#### Correlation with clinical diagnostic tests

**Figure 5:**
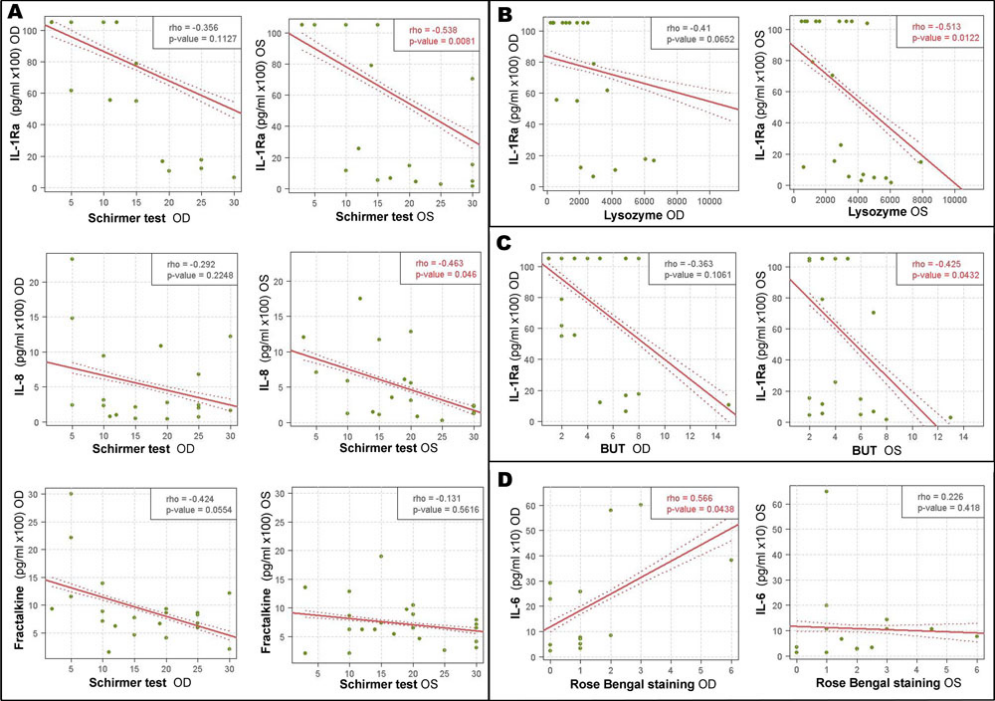
Correlation of OD and OS tear cytokine/chemokine levels with corresponding OD and OS clinical measurements of tear production and ocular surface integrity in DED patients. IL-1Ra, IL-8/CXCL8, and Fractalkine/CX3CL1 levels correlated negatively with tear production tests, and IL-6 levels correlated positively with rose Bengal staining test (measuring ocular surface integrity). **A**: Schirmer test, **B**: lysozyme levels, **C**: BUT, and **D**: Rose Bengal staining. Cytokine/chemokine values are expressed in pg/ml in a 100× (IL-1Ra, IL-8 and fractalkine) or 10× (IL-6) scale. Spearman's rho correlation coefficient was determined. Dotted lines indicate the 95% confidence bands. IL, interleukin; IL-1Ra, interleukin 1 receptor antagonist; OD, right eye; OS, left eye.

**Figure 6:**
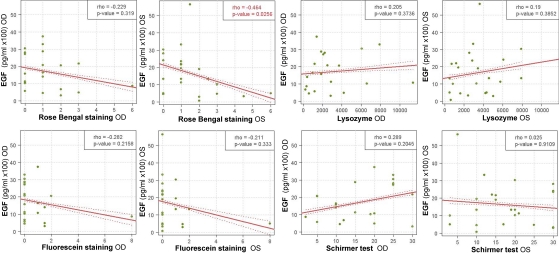
Correlation of OD and OS tear EGF with corresponding OD and OS clinical measurements of tear production and ocular surface integrity in DED patients. EGF levels correlated negatively with rose Bengal and fluorescein staining tests (measuring ocular surface integrity) and positively with Lysozyme and Schirmer tests (measuring tear production). EGF values are expressed in pg/ml in a 100× scale. Spearman ranked correlation test was used and the strength of correlation between variables was determined by the Spearman's rho correlation coefficient. EGF, epidermal growth factor; OD, right eye; OS, left eye.

Each clinical diagnostic test was correlated with each of the seven molecules detected in more than half of the moderate DED patients’ tears. Only IL-1Ra, IL-8/CXCL8, fractalkine/CX3CL1, and IL-6 showed statistical correlations or similar relevant tendencies (Figure 5). In general, there was an inverse relationship between the levels of IL-Ra, IL-6, IL-8/CXCL8 and fractalkine/CX3CL1 and the Schirmer test, lysozyme, and TBUT values. IL-6 levels in both eyes correlated positively with rose Bengal staining, but only significantly for OD (Figure 5). EGF levels were positively correlated with Schirmer and lysozyme tests, which measure tear production, and negatively correlated with rose Bengal and fluorescein staining, which measure ocular surface integrity (Figure 6). However, except for the correlation of OS EGF levels with rose Bengal staining (p=0.0256), none of these reached statistical significance.

## Discussion

In this study, we found that several cytokines and chemokines were increased in tears from evaporative-type MDG-dependent DED. These patients had mild-to-moderate signs at the ocular surface with moderate-to-severe symptoms, and there were some correlations between the inflammatory mediators and the signs and symptoms. DED is a type of ocular surface inflammation where signs and symptoms often do not correlate well, particularly in the mild-to-moderate cases [6,24-29]. There is growing interest in finding a potential biomarker(s) that reflect the symptomatology of these patients. In the more severe forms of DED, the levels of several cytokines and chemokines are increased [18,32-34,48,49], and correlation studies revealed associations between some of those inflammatory mediators with clinical parameters [30,31]. Furthermore, increases in inflammatory cytokine tear concentrations may be responsible for the irritation symptoms and ocular surface disease in DED [50]. However much less is known about the cytokine/chemokine levels in those patients having mild-to-moderate DED. Those patients are characterized by being highly symptomatic, but without important clinical sign alterations.

Our study revealed that tears from mild-to-moderate DED patients have significantly increased levels of EGF, fractalkine/CX3CL1, IL-1Ra, IP10/CXCL-10, IL-8/CXCL8, and VEGF. Previous studies by others have shown that EGF was decreased in tear fluid from Sjögren’s syndrome patients [18], and also in those patients with DED but without MGD [31]. However, Lam et al. [31] and Rao et al. [51] also showed that patients with DED and MGD, like the ones in our study, had higher EGF tear levels.

We previously have described the presence of fractalkine/CX3CL1 in normal human tears [46], and to our knowledge, this is the first report that describes increased levels of this molecule in tears from DED patients. Fractalkine/CX3CL1 differs greatly from other chemokines in that it can exit the cell either in a soluble form or in a membrane-bound form [52]. It interacts with the unique receptor CX3CR1 that is expressed on monocytes, natural killer cells, and some T cells. Soluble fractalkine/CX3CL1 is a potent chemoattractant for CX3CR1^+^ leukocytes. Fractalkine/CX3CL1-CX3CR1 interaction contributes to the development of various inflammatory diseases such as rheumatoid arthritis, asthma, Wegener’s granulomatosis, Crohn’s disease, psoriasis, glomerulonephritis, experimental autoimmune anterior uveitis, and atherosclerosis [52-57]. A role for fractalkine/CX3CL1 in a mouse model of Sjögren syndrome has been very recently proposed [19]. Its role in human dry eye is yet unknown and deserves further analysis.

IL-1Ra levels were also significantly increased in tears from patients. This is consistent with a previous report showing that IL-1Ra concentration is increased in DED tear fluid and in conjunctival cytology specimens from Sjögren’s syndrome patients with aqueous tear deficiency [32]. It is produced by corneal epithelial cells and fibroblasts [58]. IL-1Ra is a naturally occurring cytokine receptor antagonist [59,60] that serves as a modulator of immune responses regulating the agonist effects of IL-1 during chronic inflammatory and infectious diseases. IL-1Ra acts as a natural anti-inflammatory protein in arthritis, colitis, and granulomatous pulmonary disease [59].

Tears from the DED patients had significantly increased IP-10/CXCL10 levels. This molecule is a potent chemoattractant for activated Th1 and natural killer cells. IP-10/ CXCL10 binds to cells through the CXCR3 receptor, which is preferentially expressed in Th1 cells as well as in eosinophils. Significantly increased IP-10/CXCL10 expression in both corneal and conjunctival epithelium has been described in an experimental animal model of dry eye [61] and more recently in tears from Sjögren Syndrome DED patients, and also, but non significantly, in non-Sjögren DED patients [51]. Human conjunctival epithelial cells in vitro secrete significantly increased amounts of IP-10/CXCL10 when stimulated by TNF-α and INF-γ [62]. Increased expression of this molecule also occurs in some human chronic inflammatory diseases, as in the airways of subjects with chronic obstructive pulmonary disease [63] and in patients with asthma [64,65], where the level is correlated with lymphocyte number. Chemokines that preferentially attract Th1 cells via the CXCR3 receptors, such as IP-10/CXCL10 or MIG/CXCL9, are involved in the polarization of T cell recruitment as they concomitantly block the migration of Th2 cells in response to CCR3 ligands [66].

We also found that VEGF levels were increased in mild-to-moderate DED patients. VEGF expression in conjunctival biopsies and tears are increased in some ocular chronic inflammatory diseases such as vernal keratoconjuntivitis or atopic keratoconjuntivitis [67,68], where it plays a crucial role in the remodeling process of these severe allergic conjunctival disorders [68]. Conjunctival fibroblasts significantly increase VEGF mRNA expression under the effect of several cytokines, such as TGF-β1, IL-1β, and IL-4 [69]. High levels of VEGF immunoreactivity also occur in conjunctival epithelial cells and inflammatory cells in vernal keratoconjuntivitis biopsies [68]. Recent studies have also shown that, in addition to its well known angiogenic role [70], VEGF can also act as a direct proinflammatory mediator during the pathogenesis of rheumatoid arthritis [71-74].

IL-6 has been described as one of the key molecules in DED. This molecule is increased in the tears and conjunctival epithelium of DED patients [18,30,31,33,34,49], and its presence correlates with some clinical parameters in patients with severe forms of DED [30,31,34]. For this reason it has been suggested as a possible biomarker for DED. However, in our study, it was detected in only 65% of the samples, and the levels were not different from those found in controls. Additionally, we could detect IL-1β or TNF-α (that have also been described as increased in DED [30,32,34]) in only 30% and 2%, respectively, of the patients. This could be related to the fact that the patients included in this study had evaporative-type and not aqueous-tear deficient DED, with mild clinical signs. This is in agreement with previous studies in symptomatic, moderate dry eye patients, where there were no increases in levels of IL-1β, IL-6, IL-8/CXCL8, GRO-b, ICAM-1, TRAIL, or ephrin A5 in tear fluid [36]. Some of these agents are substantially increased in severe DES. In another study in which IL-1β, IL-6, and pro-matrix metalloproteinase (MMP)-9 tear levels were measured in patients with different types of ocular diseases, including a group of 20 hyposecretive moderate dry eye patients, only proMMP9 was significantly increased in DED patients [37].

Although no correlations with global scores for the SODQ and SANDE questionnaires were found for any of the cytokines, significant differences existed for IL-6 and IL-8/CXCL8 in association with pain sensation scores. Maximal values of both IL-6 and IL-8/CXCL8 were related to pain sensation score 1. Correlations of IL-6 levels with the gritty/sandy sensation score and with the blurred vision scores of 3–4 were close to statistical significance. Lam et al. [31] proposed that IL-6 levels in tears of mild level 1 DED can be related to inflammation-induced hyperalgesia, which may be the cause for irritation symptoms in these patients. DED has been referred to as a chronic pain syndrome. Pain is a very centrally mediated phenomenon and is expressed very differently in individuals. Therefore one would expect better objective correlation with signs. Cytokines play a role in pain and hyperalgesia by direct action upon nerve endings, ion channels, and through other pain mediators [75]. Proinflamatory cytokines (i.e., IL-1β, TNF-α, IL-6, IL-8/CXCL8, and fractalkine/CX3CL1) are mostly algesic, while the so-called anti-inflammatory ones (IL-10, IL-4) or cytokine antagonists (such as IL-1Ra) have analgesics properties [75-77]. An imbalance of pro- and anti-inflammatory cytokine expression, rather than individual specific cytokine levels, is of importance for individual pain susceptibility. Individual cytokine profiles may be of diagnostic importance in chronic pain states, and might guide the choice of treatment [75]. Although therapies with anti-cytokine agents are currently used effectively for the treatment of inflammatory pain conditions, inhibition of downstream signaling molecules rather than particular cytokines has been proposed as a safer therapy due to the pleiotropic and redundancy of the cytokine system [78].

Our results also showed that fractalkine/CX3CL1, IL-6, IL-8/CXCL8, IL-1Ra, and VEGF levels were inversely correlated with Schirmer test results and lysozyme levels, which measure tear production. Although not significant, EGF levels were positively correlated with meniscus height, Schirmer test, and tear lysozyme levels and negatively correlated with vital staining by fluorescein and rose Bengal, which demonstrate ocular surface damage. Although statistical significance was not reached in all these correlations, our results are in agreement with those describing the negative correlation of IL-6 with Schirmer test results [30]. We also found that IL-6 was positively correlated with rose Bengal staining (in both eyes, statistically significant for OD) and with fluorescein staining (only for OD). Thus, higher levels of this cytokine were associated with more damage to the ocular surface. Similarly, Lam et al. reported that Schirmer test scores were negatively correlated with IL-6 and IL-8/CXCL8 and positively correlated with EGF [31]. They also found that EGF levels were negatively correlated with corneal fluorescein and conjunctival lissamine green staining scores.

Except for IL-6, we found no significant differences in cytokine levels between OD and OS eyes. However, we did find differences between OS and OD when we correlated cytokine levels with the clinical signs and symptoms for each eye. This inconsistency between eyes probably relates to the high variability for each of the clinical tests. Thus, pooling collected tear samples between eyes may lead to erroneous conclusions in both clinical and research applications.

In conclusion, we have shown that in this sample of mild-to-moderate evaporative-type DED patients, where signs of tear production were normal and vital staining failed to show ocular surface damage, inflammatory mediators in tears were a more sensitive and reliable indicators of disease. Additionally, IL-1Ra, IL-6, CXCL8/IL-8 and EGF levels correlated with pain and with clinical parameters measuring tear stability, tear production, and ocular surface integrity. All these results suggest that inflammation plays a role not only in severe or aqueous-deficient DED but also in mild-to-moderate evaporative DED due to MGD.
